# Postpartum Depression Among Mothers of Infants Hospitalized in the Neonatal Intensive Care Unit During the COVID-19 Pandemic

**DOI:** 10.7759/cureus.44380

**Published:** 2023-08-30

**Authors:** Mine Ozdil

**Affiliations:** 1 Department of Pediatrics, Division of Neonatology, University of Health Sciences, Balıkesir Atatürk City Hospital, Balıkesir, TUR

**Keywords:** pandemic, postpartum depression, neonatal intensive care unit (nicu), perinatal mental health, maternal, covid-19

## Abstract

Background

Neonatal intensive care unit (NICU) hospitalization of newborn babies has been shown to have a negative impact on the mental health of postpartum women. The mental health of new mothers may be further burdened by the effects of the coronavirus disease 2019 (COVID-19) pandemic on social, economic, and psychological dimensions. This study aimed to evaluate postpartum depression and related factors in mothers of infants hospitalized in NICU during two distinct COVID-19 pandemic periods and examine any additional effects of the pandemic on the mental health of postpartum women.

Methodology

The Edinburgh Postpartum Depression Scale (EPDS) was applied to 250 NICU mothers during the COVID-19 pandemic. The first 125 women’s children were hospitalized during a period of high number of cases and deaths when restrictions were in place for NICU parental visits (November 2021 to February 2022, the early group). The remaining 125 women completed the scale when there was a lower number of cases and restrictions had been eased (March to June 2022, the late group).

Results

In the early group, the EPDS scores were statistically higher (7.53.9 vs. 5.63.4; p < 0.001), smoking and NICU stay duration were significantly higher (p = 0.01), whereas the duration of marriage was significantly lower (p = 0.01). Women in the late group with EPDS scores ≥13 were statistically less educated (p = 0.01). EPDS scores ≥13 were significantly associated with depression during pregnancy and with a history of abortion/stillbirth/neonatal death (odds ratio (OR) = 5.240, 95% confidence interval (CI) 1.114 to 27.967, p = 0.03 and OR = 1.641, 95% CI = 1.009 to 2.669, p = 0.04, respectively).

Conclusions

NICU admission is a significant maternal risk factor for postpartum depression due to the disruption of maternal-infant bonding, and this risk may be exacerbated during times of global public health crises such as the COVID-19 pandemic. Depression during pregnancy and the presence of a perinatal loss may also contribute to worse postpartum mental outcomes in NICU mothers.

## Introduction

During and after pregnancy, a variety of psychological disorders, such as postpartum blues, anxiety, posttraumatic stress disorder, and postpartum depression (PPD), can affect women [1]. Mental disorders are the most prevalent perinatal morbidity and may impair psychological and neuromotor development in babies of affected mothers, resulting in neuropsychiatric disorders and motor, social, cognitive, and language delays [2]. PPD is an underestimated, unrecognized, and undertreated problem of the postnatal period, affecting 10-30% of women [3]. Cases of PPD are known to cluster significantly within the first four weeks after childbirth [4]. Current guidelines from the American College of Obstetricians and Gynecologists and the American Academy of Pediatrics recommend routine screening for maternal PPD during well-child visits. Despite recommendations, screening is not widely practiced in healthcare settings globally [1].

PPD may be significantly more severe in mothers with infants in the neonatal intensive care unit (NICU). Between 25.5% and 62% of NICU mothers experience PPD in the first postpartum month, and the rate of suicidal ideation increases from 14% to 33% in NICU mothers compared to postpartum women in the general population [5,6]. Stressful events related to NICU admissions, such as low birth weight, preterm/urgent births, or births after a complicated pregnancy, the emotional challenges related to the infants’ acute illness, and disrupted mother-infant bonding are known to increase PPD and symptoms related to anxiety [7].

Coronavirus disease 2019 (COVID-19), declared a pandemic by the World Health Organization in March 2020, is known to have had adverse global effects on the mental health of infected and uninfected women, including pregnant and postpartum women. Fear of adverse effects of the virus and vaccines on the developing fetus; social isolation; less contact with friends, family, and social care services; and financial problems related to lockdown measures exerted an additional strain on the mental health of pregnant and postpartum women. Furthermore, restrictions during delivery, such as the requirement to wear a mask or limitations on accompaniment, and restricted prenatal and postnatal hospital visits placed postpartum women at a greater risk for PPD [8]. Moreover, cases of family violence were reported to have increased during the COVID-19 pandemic [9].

Uncertainty regarding the effects of COVID-19 infection has been a psychological burden on expectant and new mothers for a long time [10]. As the pandemic spread, pregnancy-related COVID-19 infections were associated with higher rates of maternal and neonatal morbidity, including severe infections in pregnant women, an increased risk of preterm birth, maternal deaths, and fetal mortality [11]. Furthermore, it has been shown that pregnant women with COVID-19 infection are more likely to experience premature membrane rupture, fetal distress, preeclampsia, cesarean sections, low-birth-weight babies, stillbirths, and admissions to a NICU during the pandemic [12]. The bond between mother and child, already damaged by the baby’s stay in the NICU, was further harmed by strictly prohibited visits during this psychologically vulnerable period of time [13]. Additionally, due to the bidirectional relationship between inflammation and depression in COVID-19-infected pregnant and postpartum women, their mental health may be further impacted [14]. The prevalence of psychological disorders and symptoms, such as depression and anxiety, increased significantly during the postnatal period with the combined effects of all factors.

This study aimed to screen for depressive symptoms in postpartum women whose infants were hospitalized in a tertiary NICU in two different stages of the COVID-19 pandemic in Turkey and search for any additional effects of the pandemic on the postpartum mental state of NICU mothers.

## Materials and methods

Study design and participants

This study enrolled women who gave birth at the Atatürk City Hospital, Balikesir, Turkey, and whose newborns were kept in the NICU from November 2021 to June 2022. Women who had recently given birth and were at least 18 years old, fluent in Turkish, willing to participate, and who provided written informed consent were included. Women who had recently given birth but declined to participate, had mental retardation, had a history of substance abuse, had twins, or had children with severe hypoxic-ischemic encephalopathy were excluded. A total of 250 postpartum women completed the Turkish version of the Edinburgh Postpartum Depression Scale (EPDS) after completing an information form that collected data regarding maternal sociodemographic and neonatal characteristics. Ethical approval for the study was obtained from Bandırma Onyedi Eylül University Noninvasive Clinical Research Ethics Committee in line with the principles outlined in the second Declaration of Helsinki (approval number: 2022-26).

Data collection

The scale was administered face-to-face to postpartum women at postnatal 14-30 days, primarily during the infants’ discharge. To exclude postpartum blues, which are a common and typically self-limiting condition appearing in the first two postpartum weeks, the scale was not applied in the first postpartum 14 days [15]. There were two groups of NICU mothers in the study. The first 125 respondents finished the survey between November 2021 and February 2022 (referred to as the early period), which coincided with the winter peak of COVID-19 caused by the Delta variant in our country and the detection of new cases of the Omicron variant, when there were the most confirmed cases (35.000-100,000/day) and deaths (150-250/day). Our parental visitation policy during this time was limited to once per week with participation from either parent and the country’s overall restriction measures were still in place. The second group consisted of the other 125 postpartum women. They completed the survey between March and June 2022 (considered the late period), when there were fewer cases (1500-15,000/day), fewer deaths (5-100/day), and fewer nationwide restrictions [16]. Additionally, both parents were allowed to visit the NICU three times per week.

Edinburgh Postpartum Depression Scale

A widely used screening tool for depression during pregnancy and the postpartum period is the EPDS, which was created by Cox et al. [17] and validated in Turkey by Engindeniz et al. [18]. The 10-item, four-point, Likert-type, self-report scale is effective at detecting emotional and cognitive PPD symptoms over the previous week with high sensitivity and specificity [19]. The total score is the sum of the individual scores; 30 points is the highest score that can be obtained; scores above 12 require additional evaluation and are cause for referral to a psychiatrist or psychologist. A score of 12 or higher indicates a 60% chance of having depression, while a score of 10.5 or less indicates a 96% chance of not having depression. Patients with scores of 10 to 12, which indicate slightly more severe PPD symptoms, should have their assessments repeated in two weeks and, if necessary, be referred [13]. Suicidal thoughts are covered in the final EPDS item, which should be carefully examined whether it is checked or not.

After obtaining informed consent, the EPDS was administered during parental visits to the NICU after completing a questionnaire about maternal demographic and neonatal variables, mostly at the time of discharge. Sociodemographic variables including parental age; education; maternal employment status; history of any disease; drug usage and smoking status; exercise and/or depression during pregnancy; income level; area of residence; planned/unplanned pregnancy; elementary/extended family; planned/unplanned pregnancy and emergent delivery; parity; history of stillbirth, abortion, and neonatal death in previous pregnancies; type of delivery; presence of any postpartum complications; and the amount of weight gain during pregnancy were recorded. In addition, the history of COVID-19 infection and immunization against COVID-19 during and after pregnancy were investigated. Neonatal variables including gestational age, gender, birth weight, duration of mechanical ventilation and NICU stay, and the type of feeding (breastfeeding/formula or mixed) were recorded. Women with EPDS scores ≥13 were referred for psychiatric evaluation, and those with scores between 10 and 12 were re-evaluated with telephone contact two weeks after the first survey and referred for consultation if necessary.

Data analysis

Data were analyzed using SPSS version 24 (IBM Corp., Armonk, NY, USA). Descriptive statistics were computed for demographics and main study variables. Categorical data were described as n and percentage, and numerical data as mean ± SD if normally distributed, and median (IQR) if non-normally distributed. Kolmogorov-Smirnov test was used to reveal the data distribution. Continuous variables were evaluated using the Student’s t-test or Mann-Whitney U-test depending on the distribution characteristic of the data, and categorical variables were evaluated using the chi-square or Fisher’s exact test. The cut-off value of EPDS was defined as ≥13, according to the literature [17,18]. Pearson correlations were calculated to define any relationship with EPDS scores. A p-value <0.05 was considered statistically significant. Finally, odds ratios (ORs) of multiple risk factors were evaluated by multiple logistic regression analysis with 95% confidence intervals (CIs).

## Results

In both groups, the EPDS was administered to participants on median postpartum day 18 (IQR = 16-20). Except for immunization against COVID-19 and type of delivery, maternal sociodemographic and neonatal variables were statistically similar between groups (p > 0.05). Rates of confirmed COVID-19 infection during pregnancy were comparable between the two groups (6% vs. 9%, p = 0.30); however, immunization against COVID-19 during pregnancy was significantly higher in the early group (20%, n = 30 vs. 9%, n = 13; p = 0.001). Significantly fewer normal vaginal births occurred in the early group (33% vs. 66%, p = 0.004). Table 1 and Table 2 present the sociodemographic and neonatal variables of the mothers and newborns in the two groups, respectively.

**Table 1:** Comparison of maternal demographic characteristics between the early and late groups. COVID-19: coronavirus disease 2019; IQR: interquartile range; SD: standard deviation

		Early group	Late group	P-value
		n = 125	n = 125	
Maternal age (years), median (IQR)		28 (23.5–32)	28 (24.5–33)	0.66
Paternal age (years), median (IQR)		31 (27–35)	32 (28–35.5)	0.17
Duration of marriage (years) median (IQR)		4 (1–8)	4 (2–8)	0.45
Parity	Primiparous, n (%)	45 (36%)	39 (31%)	0.38
	Multiparous, n (%)	80 (64%)	86 (69%)	
Amount of weight gain (kg), mean ± SD		11.8 ± 10.1	10.7 ± 5.2	0.81
Education level of the mother	No formal education, n (%)	-	7 (6%)	0.17
	Primary school, n (%)	39 (31%)	49 (39%)	
	Secondary high school, n (%)	50 (40.5%)	30 (24%)	
	University/doctorate, n (%)	36 (28.5%)	39 (31%)	
Education level of the father	No formal education, n (%)	-	2 (2.5%)	0.06
	Primary school, n (%)	40 (32%)	53 (42%)	
	Secondary high school, n (%)	48 (38%)	42 (33%)	
	University/doctorate, n (%)	37 (30%)	28 (22.5%)	
Income status	Low, n (%)	9 (7%)	10 (8%)	0.61
	Middle, n (%)	110 (88.5%)	104 (83.5%)	
	High, n (%)	6 (4.5%)	11 (8.5%)	
Area of residence	Urban/suburban, n (%)	112 (89.6%)	115 (92%)	0.37
	Village, n (%)	13 (10.4%)	10 (8%)	
Type of family	Elementary, n (%)	111 (89%)	114 (91%)	0.51
	Extended, n (%)	14 (11%)	11 (9%)	
Planned pregnancy, n (%)		92 (74%)	92 (74%)	0.99
Employment during pregnancy, n (%)		25 (20%)	26 (21%)	0.99
History of miscarriage/stillbirth/neonatal death, n (%)		36 (30%)	41 (33%)	0.62
History of chronic diseases, n (%)		31 (25%)	25 (20%)	0.38
COVID-19 infection during pregnancy, n (%)		7 (6%)	11 (9%)	0.30
COVID-19 immunization during pregnancy, n (%)		27 (22%)	11 (9%)	0.001
Medication during pregnancy, n (%)		17 (14%)	19 (15%)	0.59
Smoking, n (%)		31 (25%)	26 (21%)	0.73
Pre-pregnancy depression, n (%)		24 (19%)	24 (19%)	0.99
Depression during pregnancy, n (%)		16 (12.5%)	19 (15%)	0.09
Exercise during pregnancy, n (%)		29 (23%)	31 (25%)	0.34
Postpartum family support (person), median (IQR)		2 (1-2)	2 (1-2)	0.09
Emergent delivery, n (%)		49 (39.5%)	52 (42%)	0.72
Postpartum complication, n (%)		26 (21%)	31 (25%)	0.76

**Table 2:** Comparison of neonatal variables between the early and late groups. IQR: interquartile range; MV: mechanical ventilation; NICU: neonatal intensive care unit; SD: standard deviation

		Early group	Late group	P-value
		n = 125	n = 125	
Gestational age (weeks), median (IQR)		36 (33–39)	35 (34–38)	0.23
Birth weight (g), mean ± SD		2,667 ± 872	2,519 ± 765	0.20
Gender	Female, n (%)	55 (44%)	50 (40%)	0.46
	Male, n (%)	70 (56%)	75 (60%)	
Type of birth	Cesarean section, n (%)	42 (34%)	19 (15%)	0.004
	Vaginal delivery, n (%)	83 (66%)	106 (85%)	
Duration of MV (days), median (IQR)		4 (2–7)	4 (2–7)	0.96
Duration of NICU stay (days), median (IQR)		17 (16–21)	16 (15–29)	0.18
Type of feeding	Breast milk, n (%)	52 (42%)	56 (45%)	0.29
	Formula, n (%)	21 (16%)	13(10%)	
	Mixed, n (%)	52 (42%)	56 (45%)	

The mean EPDS score of the total sample was 6.6 ± 3.8. The mean EPDS score in the early group was significantly higher than the late group (7.5 ± 3.9, minimum-maximum = 3-23 vs. 5.6 ± 3.4, minimum-maximum = 2-20; p < 0.001). The percentage of women with EPDS scores ≥13 in the total sample was 11% (n = 28). In the early period, the percentage of women with EPDS scores ≥13 was 15% (n = 19), whereas it was 7% (n = 9) (p = 0.015) in the late period. The percentages of women with EPDS scores ≥10 were 19% (n = 24) and 12% (n = 15) in the early and late periods, respectively (p = 0.17). A total of 28 (10%) women were referred for psychiatric consultation after the first survey. Women with EPDS scores between 10 and 11 were evaluated by a phone call after a two-week period, and the on-call survey revealed an additional three postpartum women in each group with EPDS scores ≥13. The rate of postpartum women referred to psychiatric consultation was significantly higher in the early group when compared to the late group (18%, n = 22 vs. 9.5%, n = 12; p = 0.015).

In the statistical analysis of the early group, history of depression during pregnancy (63% vs. 37%, p < 0.001), smoking (26% vs. 4%, p < 0.001), and duration of NICU stay (18, IQR = 16-22 days vs. 16, IQR = 15-21 days; p = 0.04) were statistically higher and duration of marriage was statistically lower (2, IQR = 1-3 years vs. 5, IQR = 2-9 years; p = 0.01) in women with EPDS scores ≥13. In the late group, a history of depression during pregnancy was statistically higher in women with EPDS scores ≥13 (33% vs. 11.5%, p = 0.04), and these women were statistically lower educated (75% vs. 42%, primary and secondary vs. high school and doctorate, respectively, p = 0.01).

In the early group, the Pearson correlation tests revealed weak correlations of EPDS scores ≥13 with chronic medication use during pregnancy (r = 0.294, p = 0.002), smoking during pregnancy (r = 0.351, p < 0.001), and duration of marriage (r = 0.228, p = 0.01). In the late group, EPDS scores correlated weakly with maternal education level (r = 0.241, p = 0.01), multiparity (r = 0.202, p = 0.04), and maternal age (r = 0.223, p = 0.02). No correlation between immunization rates and EPDS scores was detected. In the total sample, EPDS scores correlated very weakly with maternal education level (r = 0.16, p = 0.01).

Multiple logistic regression analysis of the entire cohort was used to identify the variables related to EPDS score ≥13. The analysis demonstrated a significant association of EPDS score ≥13 to previous history of abortion/stillbirth and/or neonatal death with an OR of 1.641 (95% CI = 1.009 to 2.669, p = 0.04) and to depression during pregnancy with an OR of 5.240 (95% CI = 1.114 to 27.967, p = 0.03).

## Discussion

The perinatal period is already a crucial and challenging time for the mental health of childbearing women. New mothers experience additional psychological stress as a result of pandemics and the hospitalization of newborns in NICUs [8-10]. The current study, which involved 250 postpartum women, examined the prevalence of PPD in mothers of NICU-admitted children, neonatal and maternal risk factors, and any potential impact of the COVID-19 pandemic on PPD. This is the largest and first study in Turkey examining how pandemics affect PPD in NICU mothers. In our sample, early-period NICU mothers, during which there were a high number of cases, deaths, and stricter regulations, had significantly higher EPDS scores than NICU mothers during a less severe period.

The hospitalization of a newborn is regarded as a traumatic and stressful experience. The new NICU mothers face difficulties due to the uncharted and complex nature of the NICU, their lack of medical knowledge, and their attempts to recover from childbirth [19]. Due to the disruption of the bond between mother and child caused by NICU admission, new mothers may also experience guilt, sadness, worry, and a sense of loss of maternal role. These emotions contribute to and raise the risk of depression in mothers of infants who are admitted to the NICU [19]. The mean EPDS score of mothers after hospitalization of their infant to the NICU ranges between 6.5 ± 4.1 and 13.0 ± 5.2 [3,7,20], which is consistent with the current study, which found mean EPDS scores of 6.6 ± 3.8, 7.5 ± 3.9, and 5.6 ± 3.4 in the overall, early, and late groups, respectively. Depending on the EPDS cutoff values used and the gestational weeks of the infants in the study, the frequency of PPD among NICU mothers varies greatly but can be as high as 59% based on EPDS scores [21]. In our study, 12% of women with an EPDS score of ≥13 and 19% of women with a score of ≥10 had PPD symptoms, respectively.

Numerous variables, such as an unexpected preterm birth, a history of depression, the length of the baby’s stay in the NICU, the mother’s age, education level, duration of marriage, smoking, socioeconomic status, and social support can all have an impact on the severity of PPD [22]. Prematurity is a well-known risk factor for the development of PPD and anxiety in NICU mothers [22]; however, we were not able to identify any correlation between EPDS scores and gestational week and prematurity across the entire cohort in our study. The tertiary level of our NICU, where term babies with high rates of morbidity are hospitalized, may be one explanation for this finding. Yurdakul et al. [23] and Gong et al. [24] examined PPD in NICU mothers and reported that the duration of hospital stay was associated with higher EPDS scores due to hospitalization of a sick, immature infant. In line with previous research, our study found that longer NICU stays were associated with lower EPDS scores in the early group, but this relationship was not significant in the late group, likely due to COVID-19-related concerns and ineffective maternal-infant bonding with limited parental visits.

PPD risk factors include older maternal age, lower educational attainment, shorter duration of marriage, smoking, and nulliparity [22,24]. Similarly, our study found that EPDS scores were weakly correlated with smoking and shorter marriage duration in the early group and older maternal age and lower maternal education level in the late group. Paternal educational status did not significantly affect the EPDS scores in either group. Maternal immunization rates also did not seem to exert a protective effect on PPD. Although nulliparity is known to be a risk factor for PPD, multiparity was associated with PPD development in the late group. This may be attributed to the combined effects of social isolation, crowding, and lack of privacy. Contradictory findings have been reported in the literature regarding the mode of delivery as a risk factor for PPD, but it is known that PPD can develop after an emergent cesarean section [25]. Given that there was no discernible difference in the rates of emergency cesarean sections between the early and late groups and the emergent section rate was high in both groups, the higher cesarean section rate in the early period is likely due to concern over the vertical transmission of COVID-19 to the neonate. The lack of difference in breastfeeding rates among both groups can be attributed to the absence of a mother with an active infection of COVID-19 and no interruption of breastfeeding in the case of mothers with suspected infection.

In this study, the results of the entire sample from multiple logistic regression analysis showed a significant correlation between EPDS scores ≥13 and depression during pregnancy, as well as a history of abortion, stillbirth, and/or neonatal death. PPD has been shown to begin before birth in 50% of patients with prenatal depression, and PPD has a strong correlation with prenatal depression. Prenatal depression has been shown to increase the risk of PPD [26]. Women with prior pregnancy losses, such as miscarriages, stillbirths, or induced abortions, were found to have an increased risk of PPD in the study by Giannandrea et al. [27]. In a similar manner, our study showed a correlation between EPDS scores greater than 13 and a history of stillbirth, abortion, or neonatal death.

In the early stages of the pandemic, Turkey’s approach to a newborn born to a confirmed or suspected COVID-19-positive mother was much stricter, leading to additional and unnecessary hospitalization in NICUs with limited parental visits, which had an adverse effect on maternal-infant bonding and placed an additional stress on postpartum women. In the early period of this study, from November 2021 to March 2022, the COVID-19 pandemic lockdown had been lifted, but social distancing and mask use were still advised in healthcare facilities, on public transportation, and in schools. Additionally, parental visits were still limited to once per week in the NICU where this study was conducted, and only one parent could attend. In contrast, after March 4th, 2022, when confirmed COVID-19 cases and deaths had decreased, the restrictions were loosened. Mask use was restricted to medical facilities, and HES codes were no longer required to enter shopping centers, government buildings, or to board any form of public transportation. Additionally, both parents were able to make three visits per week to our NICU [16]. It has been established that the COVID-19 pandemic impair the quality of prenatal, natal, and postnatal care. Women who gave birth during the COVID-19 pandemic experienced stress, anxiety, and depressive symptoms during pregnancy, labor, and the postpartum period. Postnatal depression rates were shown to differ from those before the pandemic due to the effects of social isolation and quarantine brought on by lockdown measures, disrupted skin-to-skin contact soon after birth, prolonged baby-mother separation due to COVID-19 infection in the mother, and feeling alone both during and after birth [28]. According to a review by Chimelewska et al. [28], during the COVID-19 pandemic, maternal deaths increased and the rate of stillbirths was shown to be higher, particularly in low- and middle-income countries. In the same study, mean EPDS scores, postnatal depression, anxiety, or both were reported to be higher during versus before the pandemic [28]. Similar studies conducted globally showed that postpartum women had higher EPDS scores during the COVID-19 pandemic [29]. On the other hand, Pariente et al. [30] reported that having a baby during the COVID-19 pandemic was associated with a lower risk of PPD, which was attributed to more family support during the quarantine and a shorter hospital stay in the postnatal ward. Similar to the majority of the literature, the study’s early period, during the disease’s peak in the nation with the highest number of cases and fatalities, saw higher rates of PPD symptoms and mean EPDS scores. A cut-off value of EPDS score >10 showed 19% and 12% PPD in the early and late period of the COVID-19 pandemic, respectively.

To our knowledge, this is the largest study to assess PPD in NICU mothers during the COVID-19 pandemic in our country. However, there are some limitations of the study. First, the study was conducted in the only tertiary care NICU unit of our city, which reflects the psychological status of mothers whose neonates have more severe problems. There can be regional/institutional variations in PPD rates and severity which may not represent the other regions of the country. Another limitation is that the follow-up of the mothers was not extended beyond two months of age. Lastly, lack of data regarding the pre-COVID-19 state and psychological assessment during pregnancy are other limitations.

## Conclusions

The study findings indicated that the COVID-19 pandemic severity could exert a negative effect on maternal mental well-being among postpartum women whose infants were hospitalized. Every new mother is at risk for PPD, but those carrying the burden of hospitalization of their newborn to the NICU are at the greatest risk and should be screened. Globally, crises in public health, such as the COVID-19 pandemic, have devastating physical, economic, social, and psychological effects. During the COVID-19 pandemic winter wave, the mental health of NICU mothers was negatively impacted, posing psychological challenges during pregnancy and the postpartum period. Doctors and nurses in NICUs should be aware of the development of PPD in this high-risk and vulnerable population, and screening for PPD, which can persist long after discharge, should be performed routinely. NICU mothers experiencing psychological distress, especially during global health events such as the COVID-19 pandemic, should be offered emotional support and referred for psychological consultation.
